# Descriptions of two new species of the genus *Colocasiomyia* (Diptera, Drosophilidae) breeding on *Rhaphidophora* host plants in Yunnan, China

**DOI:** 10.3897/zookeys.968.56677

**Published:** 2020-09-16

**Authors:** Run-Jie Jiao, Li-Hong Bai, Jian-Jun Gao

**Affiliations:** 1 Yunnan Key Laboratory of Plant Reproductive Adaptation and Evolutionary Ecology, School of Ecology and Environmental Science, Yunnan University, Kunming, Yunnan 650500, China; 2 Laboratory of Ecology and Evolutionary Biology, Yunnan University, Kunming, Yunnan 650500, China; 3 Huanglianshan National Nature Reserve, Lüchun, Yunnan 662500, China

**Keywords:** DNA barcoding, key to species, morphology, neighbor-joining tree, taxonomy

## Abstract

The genus *Colocasiomyia* de Meijere (Diptera, Drosophilidae) is known to include 30 described and nearly 60 undescribed species classified into six species groups. Among these, the *C. gigantea* group of seven known species (two Southeast Asian and five Chinese) proved to be peculiar for its specificity on monsteroid (subfamily Monsteroideae, family Araceae) host plants. In this paper, two new species, *C. todai* Jiao & Gao, **sp. nov.** and *C. liae* Jiao & Gao, **sp. nov.**, are described as members of the *C. gigantea* group with specimens collected from inflorescences of the monsteroid host species *Rhaphidophora peepla* (Roxb.) Schott and *R. crassicaulis* Engl. & Krause, respectively, in Yunnan, China. The two new species are delimitated, in comparison with all known species, based on not only morphological but also DNA barcode (partial sequence of the mitochondrial *COI*, i.e., cytochrome *c* oxydase subunit I, gene) data. A revised key to all the nine species of the *C. gigantea* species group is provided.

## Introduction

**Figure 1.**
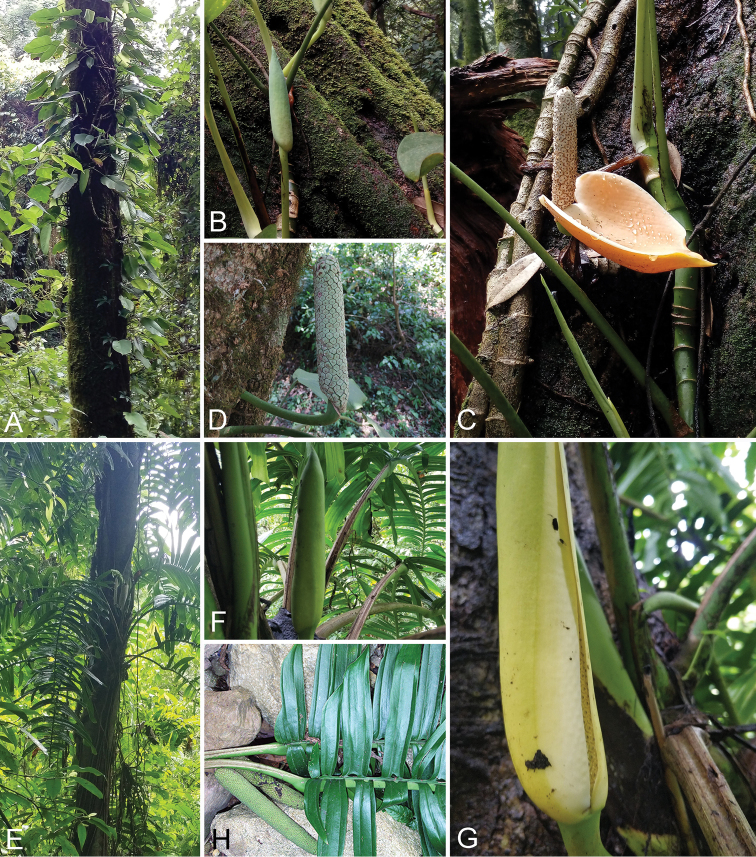
Two host plant species of *Colocasiomyia* flies **A–D***Rhaphidophora peepla* (Roxb.) Schott (Ertaipo, Mt. Gaoligongshan, Yunnan, China) **E–H***R. crassicaulis* Engl. & Karause (Qimaba, Lüchun, Yunnan, China) **A, E** plants climbing on tall trees **B, F** inflorescence buds **C, G** inflorescences **D, H** infructescences (with leaves shown together in **H**).

The genus *Colocasiomyia* de Meijere, 1914 is among a few well known anthophilic genera in the family Drosophilidae (Brncic 1983; Grimaldi et al. 2003; Fu et al. 2016). Species in this genus (30 described and nearly 60 undescribed ones) are all discovered from tropical and subtropical regions of the Old World, and taxonomically classified into six species groups: i.e., the *C. crassipes* group of two (2 described + 0 undescribed) species associated with hosts from the family Magnoliaceae, the *C. zeylanica* group of six (2 + 4) species associated with hosts from the family Arecaceae, three species groups [*C. toshiokai* group of six (6 + 0) species, *C. baechlii* group of thirty (2 + 28) species, and *C. cristata* group of thirty three (11 + 22) species] associated with aroid hosts from the subfamily Aroideae, and the *C. gigantea* group of seven known species associated with aroid hosts from the subfamily Monsteroideae (e.g., Sultana et al. 2006; Fartyal et al. 2013; Li et al. 2014; Shi et al. 2019). The *C. gigantea* group was erected by Fartyal et al. (2013) for three species: *C. gigantea* (Okada) using *Epipremnum pinnatum* in Java, Indonesia and Solomon Is., *C. rhaphidophorae* Gao & Toda using *Rhaphidophora hookeri* in Yunnan, southwestern China, and *C. scindapsae* Fartyal & Toda using *Scindapsus coriaceus* in Sabah, Malaysia. Li et al. (2014) subsequently described four additional species, i.e., *C. longifilamentata* Li & Gao, *C. longivalva* Li & Gao, *C. hailini* Li & Gao and *C. yini* Li & Gao from western Yunnan with specimens collected from inflorescences of *Rhaphidophora decursiva* (Roxb.) Schott. Here we add two new, Chinese species, i.e., *C. todai* Jiao & Gao, sp. nov. and *C. liae* Jiao & Gao, sp. nov., to the *C. gigantea* group, with specimens collected from inflorescences of *Rhaphidophora peepla* (Roxb.) Schott and *R. crassicaulis* Engl. & Krause, respectively, in Yunnan (Fig. 1).

## Material and methods

### Specimens and morphological observation

*Colocasiomyia* specimens were collected in western (Baoshan) and southern (Lüchun) Yunnan using an insect net (for adults) or by dissecting host inflorescences or infructescences (for eggs or dormant larvae within egg capsules on host infructescences), and immediately preserved in 70% (for morphological observation) or 100% (for DNA sequencing) ethanol (Table 1).

**Table 1.** *Colocasiomyia* specimens (noted in cases of egg or larval stage) used for DNA sequencing in the present study.

Species	Voucher specimens (voucher #/Stage/Gender) and collection data
*C. hailini*	01518−01522/larva/-, 01727−01731/egg/-, 01295/egg/-, 01296/egg/-; Laomengshan, Baihualing, Baoshan, Yunnan, China; *ex.* spadices or spathes of *R. decursiva*
*C. liae* sp. nov.	10485/adult/♂, 10486/adult/♀; Qimaba, Lüchun, Yunnan, China; *ex.* inflorescences of *R. crassicaulis*
	09562−09267/adult/♂, 09568−09272/adult/♀; Qimaba, Lüchun, Yunnan, China; reared from infructescences of *R. crassicaulis*
*C. longifilamentata*	01133/adult/♂, 01252/egg/-, 0158/egg/-, 01588/larva/-; Laomengshan, Baihulaling, Baoshan, Yunnan, China; *ex.* inflorescences of *R. decursiva*
*C. longivalva*	01722/adult/♀; Laomengshan, Baihulaling, Baoshan, Yunnan, China; *ex.* inflorescences of *R. decursiva*
	10103/adult/♂, 10111/adult/♂, 10114/adult/♂, 10120/adult/♀, 10121/adult/♂, 10124/adult/♀, 10127/adult/♂, 10132/adult/♀, 10133/adult/♂, 10134/adult/♀, 10135/adult/♀, 10139/adult/♂, 10143/adult/♀, 10145/adult/♀, 10146/adult/♀; Ertaipo, Baihualing, Baoshan, Yunnan, China; *ex.* inflorescences of *R. peepla*
*C. todai* sp. nov.	10100/adult/♀, 10102/adult/♀, 10105−10110/adult/♀, 10112/adult/♀, 10113/adult/♀, 10115−10118/adult/♂, 10122/adult/♂, 10128/adult/♀, 10129/adult/♂, 10130/adult/♀, 10131/adult/♂, 10136−10138/adult/♀, 10140−10142/adult/♀, 10144/adult/♀; same collection data as above
*C. yini*	10123/adult/♂; same collection data as above

We observed external morphology and detailed structures of dissected organs following the methods in Fartyal et al. (2013) and Li et al. (2014). For species illustration, we used a DinoLite Digital Eyepiece Camera to photograph the entire body, the wing, foreleg, and male/female genitalia for representative specimens. We followed McAlpine (1981) for the morphological terminology and Zhang and Toda (1992) for the definitions of measurements and indices. The type specimens are deposited in Kunming Natural History Museum of Zoology, Kunming Institute of Zoology, Chinese Academy of Sciences, Kunming, China (**KIZ**).

### Species delimitation

Adult *Colocasiomyia* specimens, either newly obtained or collected previously, were first identified and sorted into species of the *C. gigantea* group in light of morphology. DNA barcodes (i.e., the 658-bp barcoding region of the mitochondrial cytochrome *c* oxydase I, *COI*, gene) were determined for representing specimens (adults, eggs, or larvae) of each morpho-species (Table 1). We followed the methods of Li et al. (2014) for DNA extraction, PCR amplification and DNA sequencing, using Folmer et al.’s (1994) primer pair LCO1490 (5'- GGTCA ACAAA TCATA AAGAT ATTGG -3') and HCO2198 (5'- TAAAC TTCAG GGTGA CCAAA AAATC A -3'). Sequences obtained were edited in the SeqMan module of the DNAStar package version 7.1.0 (DNAStar Inc., Madison, WI). The newly determined sequences were then aligned with 45 previously determined barcodes of the *C. gigantea* group (Table 1) using the software MEGA7 (Kumar et al. 2016). Neighbor-joining (NJ) trees were constructed in MEGA7 with the sequence alignment based on *p*-distances and also Kimura 2-parameter (K2P) divergences for comparison, with node supports (bootstrap percentages) calculated with 1000 replicates. The intra- and interspecific *p*- and K2P-distances were calculated for all the species in MEGA7, and then the barcoding “gap” (Meyer and Paulay 2005) was evaluated with intra- and interspecific *p*-distances, following the methods in Meier et al. (2006, 2008).

## Results

### DNA barcoding

A total of 60 adult specimens of the *C. gigantea* group were morphologically sorted into five species, including three known (*C. longifilamentata*, *C. longivalva*, and *C. yini*) and two new ones (*C. todai* sp. nov. and *C. liae* sp. nov.) (Table 1). *COI* barcodes were determined for all 60 adults and 15 immature specimens (12 of *C. hailini*, 3 of *C. longifilamentata*) (Table 1). The alignment of the 75 newly determined barcodes (GenBank accession numbers: MT916851–MT916925) and the 45 previously determined ones spans 658 (494 conserved, 164 variable including 154 parsimony-informative) nucleotide sites.

**Figure 2.**
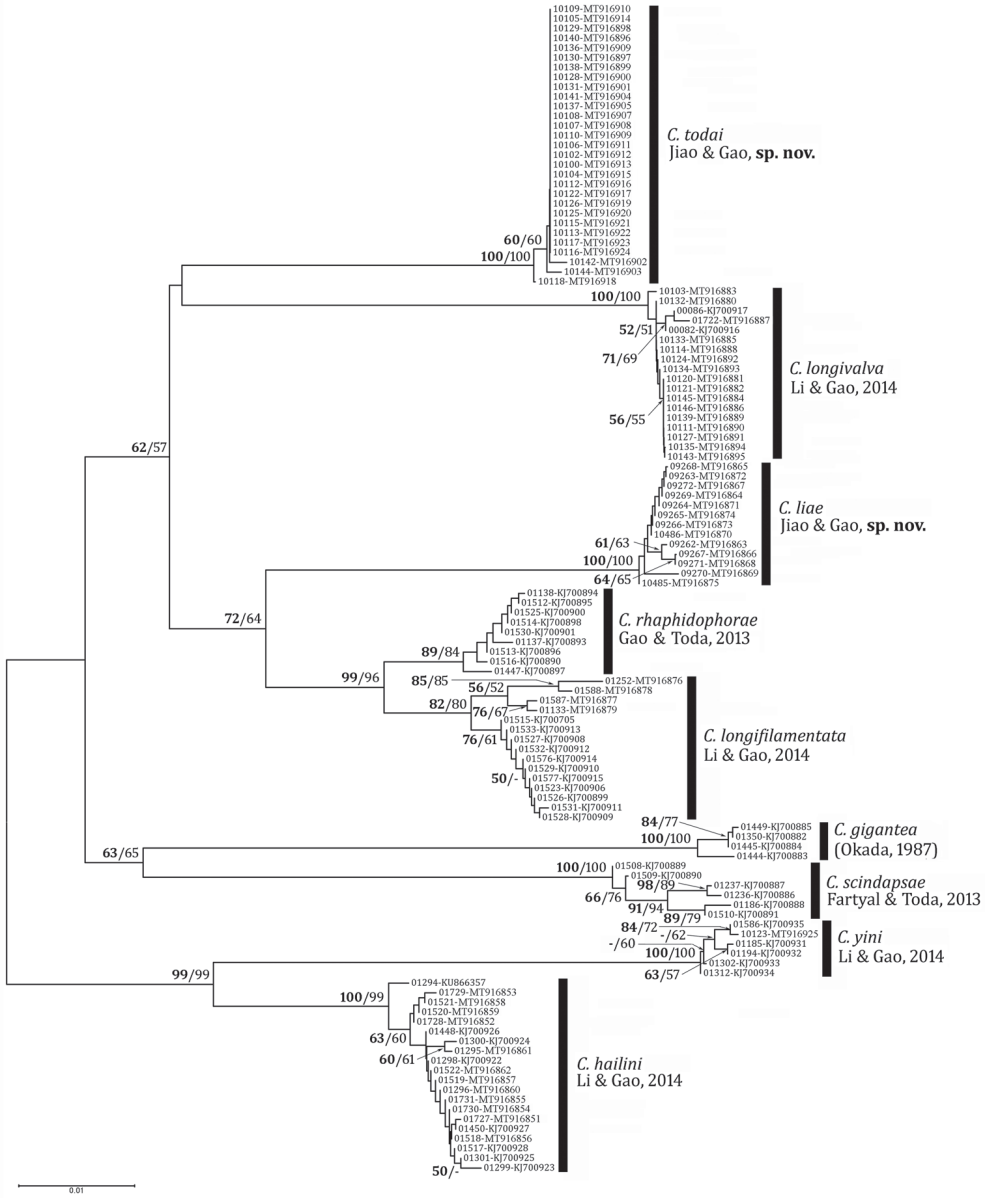
Unrooted neighbor-joining tree of the *C. gigantea* species group built based on *p*-distances between *COI* sequences. Label of each operational taxonomic unit (OTU) is given in the form of “voucher number-GenBank accession number”. Numbers beside nodes are bootstrap percentages (shown when ≥ 50; BP based on *p*-distance/BP based on K2P-distance).

The two NJ trees based on *p*- and K2P-distances showed the identical topology, but slightly differed in terms of BP: the tree based on *p*-distances yielded overall higher BPs (Fig. 2; but the K2P-distance tree not shown), confirming Srivathsan and Meier’s (2012) observation that *p*-distance performs better than K2P-distance in NJ-tree construction for DNA barcoding. The sequence clusters corresponding to the morpho-species are all compact and supported with high BPs (≥ 80 in both of the *p*- and K2P-distance methods). The grouping of two small-bodied species, *C. hailini* and *C. yini*, was strongly supported (BP = 99 in both methods). In addition, the sister-relationship between *C. rhaphidophorae* and *C. longifilamentata* was strongly supported (BP = 99 and 96, respectively).

Table 2 shows the intra- and interspecific *p*-distances in the *C. gigantea* group. The observed maximal intraspecific distance (0.0130 in *C. longifilamentata*) was the same as the minimal interspecific one (0.0130 between *C. longifilamentata* and *C. rhaphidophorae*). There was a gap (size = 0.0117 *p*-distance) between the overall mean intraspecific (0.0013) and the minimal interspecific *p*-distances, while by deleting the 5% largest intraspecific and the 5% smallest interspecific distances, a wider gap (size = 0.0491 *p*-distance) was observed.

**Table 2.** Intra- and interspecific *p*-distances in the *Colocasiomyia gigantea* species group.

#	**Species name**	**Number of sequences**	**Intraspecific distances ([Minimal, Maximal], Mean** ± **SE^a^)**	**Interspecific distances^b^**								
				**1**	**2**	**3**	**4**	**5**	**6**	**7**	**8**	**9**
1	*C. gigantea*	4	[0.0000, 0.0058], 0.0029 ± 0.0016		0.0898	0.1020	0.0940	0.1120	0.0912	0.0890	0.0907	0.1170
2	*C. hailini*	20	[0.0000, 0.0065], 0.0017 ± 0.0007	0.1006		0.0807	0.0802	0.0904	0.0797	0.0846	0.0768	0.0560
3	*C. liae* sp. nov.	13	[0.0000, 0.0063], 0.0015 ± 0.0008	0.1178	0.0912		0.0857	0.0822	0.0802	0.0923	0.0715	0.0938
4	*C. longifilamentata*	15	[0.0000, 0.0130], 0.0047 ± 0.0015	0.1081	0.0975	0.0990		0.0706	0.0130	0.0802	0.0595	0.1057
5	*C. longivalva*	18	[0.0000, 0.0049], 0.0006 ± 0.0003	0.1186	0.1037	0.0952	0.0796		0.0706	0.0822	0.0711	0.1174
6	*C. rhaphidophorae*	9	[0.0000, 0.0053], 0.0017 ± 0.0008	0.0985	0.0912	0.0978	0.0268	0.0796		0.0857	0.0579	0.1017
7	*C. scindapsae*	6	[0.0000, 0.0100], 0.0059 ± 0.0021	0.1042	0.1053	0.1090	0.0978	0.0952	0.0990		0.0819	0.1177
8	*C. todai* sp. nov.	29	[0.0000, 0.0030], 0.0003 ± 0.0002	0.0983	0.0896	0.0791	0.0729	0.0785	0.0658	0.1058		0.1057
9	*C. yini*	6	[0.0000, 0.0000], 0.0016 ± 0.0010	0.1217	0.0657	0.1047	0.1217	0.1258	0.1141	0.1342	0.1139	

^a^ SE, standard error. ^b^ Maximal values below diagonal, minimal values above diagonal.

### Taxonomy

#### Colocasiomyia gigantea

Taxon classificationAnimaliaDipteraDrosophilidae

species group Fartyal et al. (2013)

E6C7C0E4-20B0-50E8-8FC7-7B875FDFB58C

##### Included species.

*C. gigantea* (Okada, 1987); *C. rhaphidophorae* Gao & Toda and *C. scindapsae* Fartyal & Toda in Fartyal et al. (2013); *C. hailini* Li & Gao, *C. longifilamentata* Li & Gao, *C. longivalva* Li & Gao, and *C. yini* Li & Gao in Li et al. (2014); *C. todai* Jiao & Gao, sp. nov. and *C. liae* Jiao & Gao, sp. nov.

### Key to species of the *C. gigantea* species group

This key is updated from that of Li et al. (2014), referring to some figures in Okada (1987), Fartyal et al. (2013), and Li et al. (2014) which are indicated with the subscripts “O87”, “F13”, and “L14”, respectively.

**Table:** 

1	Aedeagus not pubescent; aedeagal apodeme as long as or longer than aedeagus (fig. 4C_F13_, fig. 40_L14_, fig. 47_L14_)	**2**
–	Aedeagus pubescent (except for *C. liae* sp. nov.); aedeagal apodeme distinctly shorter than aedeagus.	**4**
2	Foreleg tarsomere II with seven or eight pegs (fig. 4B_F13_). Epandrial apodeme medially narrower than epandrium (fig. 4C_F13_). Aedeagal apodeme much longer than aedeagus (fig. 4C_F13_). Distal, narrow part of oviscapt much shorter than proximal, broad part, apically shaped like arrowhead, with a pair of stout, peg-like ovisensilla at apex (fig. 4B_F13_)	***C. scindapsae* Fartyal & Toda**
–	Foreleg tarsomere II with six pegs (fig. 14_L14_, fig. 15_L14_). Epandrial apodeme well developed into distally tapering, triangular extension strongly projected anteriad, twice as long as epandrial width (fig. 38_L14_, fig. 45_L14_). Aedeagal apodeme slightly longer than aedeagus (fig. 40_L14_, fig. 47_L14_). Distal, narrow part of oviscapt rod-shaped, slightly shorter than proximal, broad part, with only trichoid ovisensilla (fig. 43_L14_, fig. 50_L14_)	**3**
3	Wing C3F index < 2/3. Distance between antennal sockets same as socket width. Distal, narrow part of oviscapt constricted subbasally on dorsal margin (fig. 43_L14_)	***C. hailini* Li & Gao**
–	Wing C3F index > 2/3. Distance between antennal sockets larger than socket width. Distal, narrow part of oviscapt finger-like, not constricted subbasally on dorsal margin (fig.50_L14_).	***C. yini* Li & Gao**
4	Labellum with 14 pseudotracheae per side. Distal, narrow part of oviscapt broadly truncate apically, much shorter than proximal, broad part (fig. 1F_O87_, fig 2H_F13_).	***C. gigantea* (Okada)**
–	Labellum with 17 or more pseudotracheae per side. Distal, narrow part of oviscapt not or only slightly truncate apically, ca 2/5 or longer the length of proximal, broad part.	**5**
5	Epandrium notched above basal corner of epandrial ventral lobe (fig. 30_L14_). Ventral lobe apically with a grooved, finger-like peg (fig. 32_L14_). Distal, narrow part of oviscapt twice as long as proximal, broad part (fig. 36_L14_)	***C. longivalva* Li & Gao**
–	Epandrium not notched along posterior margin. Ventral lobe apically with an ungrooved, inward-curved peg or thick spine. Distal, narrow part of oviscapt as long as or shorter than proximal, broad part.	**6**
6	Labellum with 17 pseudotracheae per side. Epandrium broad, with short ventral lobe apically inlaid with thick, long, inward-curved, spine-like seta (Fig. 4A, B)	***C. todai* Jiao & Gao, sp. nov.**
–	Labellum with ≥ 21 pseudotracheae per side. Epandrium with long ventral lobe apically inlaid with short peg.	**7**
7	Epandrium with setae on posterior margin in addition to those on ventral lobe (fig. 24_L14_). Distal, narrow part of oviscapt nearly as long as proximal, broad part (fig. 28_L14_)	***C. longifilamentata* Li & Gao**
–	Epandrium with no setae on posterior margin above ventral lobe (fig. 3C_F13_, Figs 5A, B); distal, narrow part of oviscapt distinctly shorter than proximal, broad part (fig. 3G_F13_, Fig. 5F)	**8**
8	Labellum with 21−22 pseudotracheae per side. Ventral lobe of epandrium well developed, narrowing distally in lateral view, scabbard-like, with 3 long setae on dorsosubbasal margin, apically inlaid with a short peg; cercus with slightly projected ventral apex (fig. 3C_F13_). Aedeagal apodeme ca 3/4 the length of aedeagus (fig. 3D_F13_). Distal, narrow portion of oviscapt narrowing distally, gently curved dorsad (fig. 3G_F13_)	***C. rhaphidophorae* Gao & Toda**
–	Labellum with 34 pseudotracheae per side. Ventral lobe of epandrium prolonged like a rod, distally slightly broadened in lateral view, with 2 long and 1 medium-length setae on its insertion, apically inlaid with a relatively long, claw-like peg; circus ventrally lacking projected apex (Fig. 5A, B). Aedeagal apodeme short than 1/2 the length of aedeagus (Fig. 5D, E). Distal, narrow portion of oviscapt extended with nearly even width, slightly sinuate (Fig. 5F).	***C. liae* Jiao & Gao, sp. nov.**

### Descriptions of species

#### Colocasiomyia todai

Taxon classificationAnimaliaDipteraDrosophilidae

Jiao & Gao sp. nov.

DB5EDBCB-C320-57B6-966C-F43B4C9FD2D6

http://zoobank.org/A34F80B7-8EB2-4686-BE1F-54AD9364A8CF

Figures 3A–E , 4

##### Diagnosis.

This species closely resembles *C. longivalva* in external morphology and structure of male genitalia, but can be distinguished from the latter by epandrium with short ventral lobe apically inlaid with a thick, long, inward-curved, spine-like seta (Fig. 4A, B); surstylus with 2 small, peg-like sensilla on inner, apical surface, 2 tiny, tooth-like setae on inner, ventrosubapical surface, and 1 tiny, trichoid seta on inner, dorsosubapical surface (Fig. 4C); and distal, narrow portion of oviscapt shorter than proximal, broad portion (Fig. 4F).

##### Description.

(♂, ♀). ***Head***: Supracervical setae about 17 per side. Dorsomedial arm of tentorial apodeme about 2/3 as long as dorsolateral arm. Eye red, somewhat roundish, lacking interfacetal setulae. Frontal vitta mat, black. First flagellomere not concave on inner margin. Facial carina trapeziform, medially twice as wide as first flagellomere, as long as pedicel and first flagellomere combined. Palpus convex on ventrodistal portion. Cibarium posterior sensilla minute, 1 or 2 per side. Labellum with 19 pseudotracheae per side.

**Figure 3.**
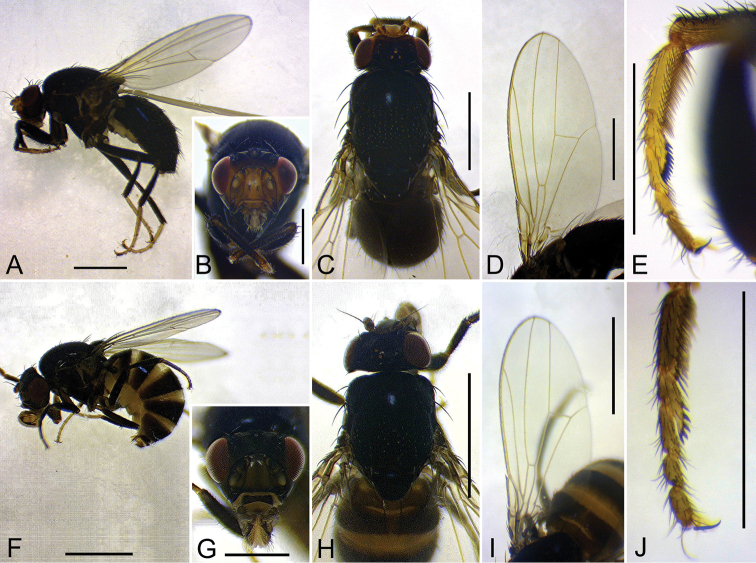
Adult males of the new species: lateral habitus, head (anterior view), head and thorax (dorsal view), wing (ventral view of left one in **D** dorsal view of right one in **I**), and fore leg (right one, inner view) **A–E***Colocasiomyia todai* Jiao & Gao, sp. nov. (#10122) **F–J***C. liae* Jiao & Gao, sp. nov. (#10485). Scale bars: 1.0 mm except for **B**, **E**, **G** and **J** (0.5 mm).

***Thorax*** (Fig. 3A, C): Scutum, scutellum and thoracic pleura glossy, blackish brown to black. Acrostichal setulae in 6 rows.

***Wing*** (Fig. 3D) hyaline, veins yellow. Halter grayish brown except for grayish yellow stalk.

***Legs*** (Fig. 3A, E) blackish brown to black except for grayish yellow tarsi. Foreleg second tarsomere with 10−12 pegs. Foreleg coxa large, with 1−2 long setae on underside near attachment to trochanter. Small preapical dorsal seta present only on hindleg tibia.

***Abdomen*** (Fig. 3A): Tergites glossy, entirely black; II to VI+VII each bearing setulae and setae in approximately 3−4 transverse rows; setae of posteriomost row largest. Sternites yellowish brown to blackish brown; VI somewhat triangular, posteriorly not bilobed.

**Figure 4.**
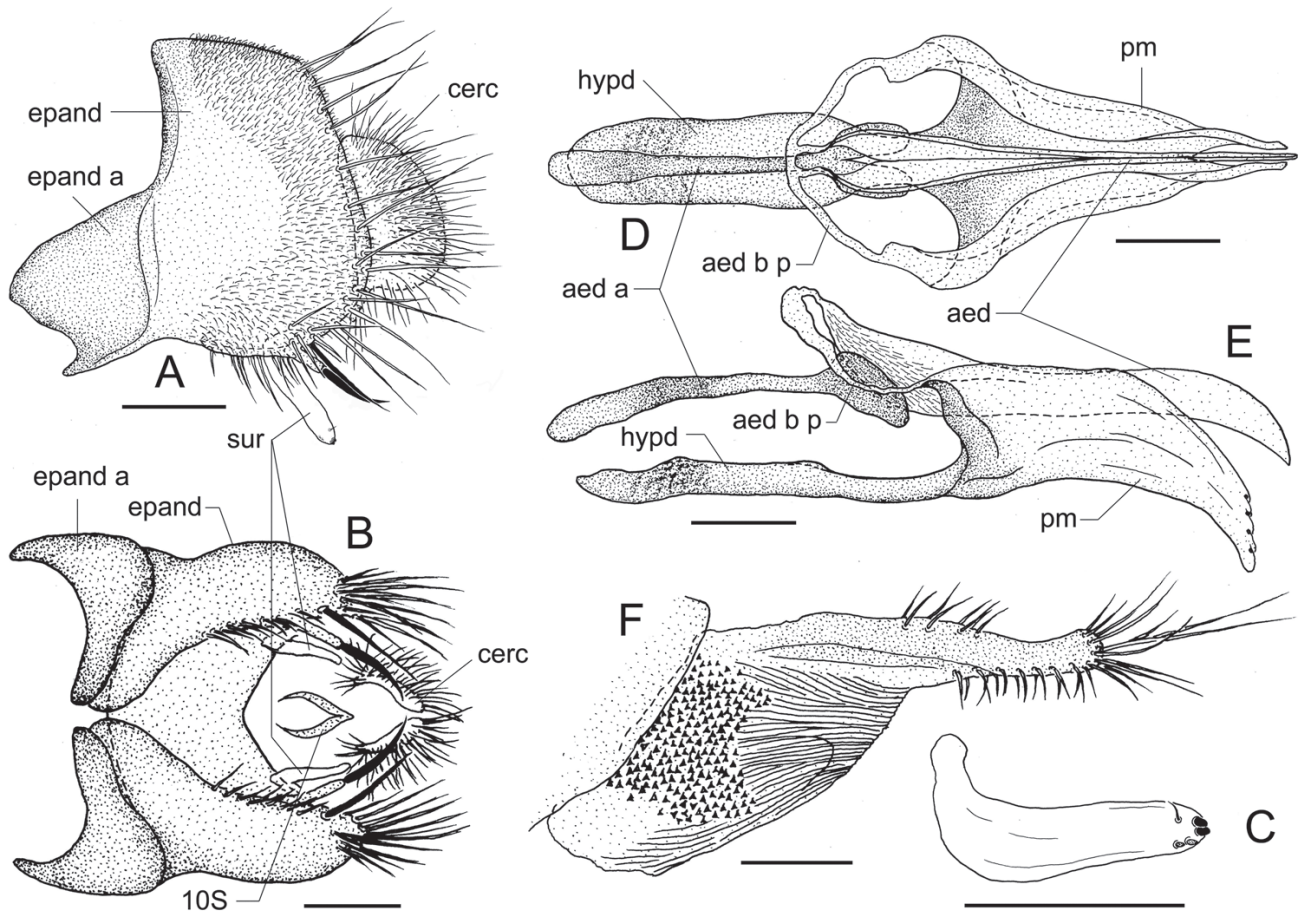
*Colocasiomyia todai* Jiao & Gao, sp. nov. Adult male (holotype #10122) and female (paratype, #10100) from Ertaipo, Gaoligong Mountains, Baoshan, Yunnan, China **A** periphallic organs (lateral view) **B** periphallic organs (ventral view) **C** surstylus (right one, inner view) **D** phallic organs (dorsal view) **E** phallic organs (lateral view) **F** oviscapt (lateral view). Abbreviations: aed = aedeagus, aed a = aedeagal apodeme, aed b p = aedeagal basal process, cerc = cercus, epand = epandrium, epand a = epandrial apodeme, hypd = hypandrium, pm = paramere, 10S = tenth sternite. Scale bars: 0.1 mm.

***Male terminalia*** (Fig. 4A−E): Epandrium broad, with large, prominent apodeme lobe on anteromedial to subventral margin, pubescent except for anterior and ventral margins; anteroventral portion curved inward, apically articulated to lateral arm of hypandrium (Fig. 4A, B). Cercus semilunar, narrowly projected at ventral apex, pubescent except for anterior margin and ventral 1/3, with ca 52 setae (Fig. 4A). Surstylus entirely narrow sclerite, elongated downward, basally articulated with epandrial ventral lobe (Fig. 4A−C). Tenth sternite less sclerotized, folded into two lateral lobes caudodorsally connected with each other (Fig. 4B). Hypandrium long, thin plate, distal 1/3 constricted, posteriorly T-shaped, with lateral arms fused to aedeagal basal processes (Fig. 4D, E). Paramere broad sword-shaped in lateral view, coalescent to hypandrium, triangular in ventral view, distally curved ventrad, with ca 4 sensilla arranged in a row (Fig. 4D, E). Aedeagus separated into a pair of lobes ventrally connected with each other, pubescent basally, subapically bent ventrad, pointed at apex; aedeagal basal processes somewhat membranous, connecting dorsobasal corners of aedeagus and lateral arms of hypandrium (Fig. 4D, E).

***Female terminalia*** (Fig. 4F): Tergite VII mid-dorsally not constricted; VIII pubescent nearly entirely, with 3 setae in a vertical row on discolored, posteroventral portion. Oviscapt with distal, narrow elongation in addition to proximal, broad portion; proximal portion with large patch of dense, distinct warts; distal portion apically more or less truncated, with ca 3−4, 7−8 and 6 trichoid ovisensilla per side on basal 2/5 of dorsal margin, entire ventral margin, and at apex, respectively, but lacking any peg-like ovisensillum.

***Measurements***: BL = 3.53 (range in 5♂ paratypes: 3.27−3.38; range in 5♀ paratypes: 3.25−3.70) mm, ThL = 1.67 (1.56−1.72; 1.49−1.73) mm, WL = 3.38 (3.09−3.45; 3.15−3.45) mm, WW = 1.50 (1.25−1.48; 1.30−1.50) mm.

***Indices***: arb = 0/0 (5♂, 5♀, or less if noted, paratypes: 0/0), FW/HW = 0.56 (0.56−0.59), ch/o = 0.59 (0.45−0.57), prorb = 0.87 (0.75−0.99), rcorb = 0.38 (0.35−0.49), orbito = 0.68 (0.64−0.95), vb = 0.46 (0.34−0.49), dcl = 0.51 (4♂, 5♀: 0.49−0.56), dcp = 1.09 (4♂, 5♀: 1.02−1.19), sterno = 0.97 (0.75−0.98), sctl = 0.75 (4♂, 5♀: 0.73−0.84), sctlp = 1.47 (1.28−1.47), C = 1.82 (1.82−2.11), 4c = 1.26 (1.05−1.25), 4v = 2.02 (1.76−2.04), 5x = 0.91 (0.89−1.06), ac = 3.45 (3.09−3.82), M = 0.46 (0.38−0.48), C3F = 0.80 (0.73−0.85).

##### Material examined.

***Holotype*** ♂ (#10122): China: *ex* inflorescence of *Rhaphidophora peepla* (Roxb.) Schott, Ertaipo, Gaoligong Moutains, Baoshan, Yunnan, China, 25°18.0'N, 98°47.0'E, ca 2200 m, 31.vii.2019, Jian-Jun Gao and Xue-Lin Ye (KIZ). ***Paratypes***: same data as holotype except for ca 2000–2250 m (5♂: #10115–18, #10131; 13♀: #10100, #10102, #101004, #10106–08, #10110, #10112, #10113, #10125, #10126, #10128, #10130) (KIZ).

##### Distribution.

China (Yunnan).

##### Host plant.

*Rhaphidophora peepla* (Roxb.) Schott (Fig. 1A–D).

##### Etymology.

Patronym, in honor of Professor Masanori J. Toda (Hokkaido University), who dedicated himself to the studies of taxonomy and flower-visiting/breeding behaviors of *Colocasiomyia* flies.

##### Remarks.

Li et al. (2014) described *C. longifilamentata*, *C. hailini*, *C. yini* and *C. longivalva* with specimens collected exclusively from inflorescences of *R. decursiva* at Baihualing, Baoshan, Western Yunnan, but mentioned that very few adults of the last two species, especially *C. longivalva*, were collected from inflorescences of *R. decursiva*. Our subsequent field work there has revealed that *C. longifilamentata* and *C. hailini*, rarely together with *C. yini*, share inflorescences/infructescences of *R. decursiva* as their breeding resources but that *C. longivalva* does not breed on this plant at all (data not shown). Recently, we have found that *C. longivalva*, together with *C. todai* sp. nov., use *R. peepla* as a host plant: adults of both species were abundantly collected from inflorescences of this plant (Table 1); and a large number of *Colocasiomyia* 1^st^-instar larvae were found overwintering within egg capsules between growing pistils of infructescences, and they were later identified as *C. longivalva* or *C. todai* sp. nov. by DNA barcoding or by examining the morphology of adults obtained from rearing the 1^st^-instars (data not shown).

#### Colocasiomyia liae

Taxon classificationAnimaliaDipteraDrosophilidae

Jiao & Gao sp. nov.

DAA0938C-94CC-5C94-9EBE-07FF7C60A04E

http://zoobank.org/D074B28A-894D-4B01-9D8E-537EE4140D29

Figures 3F–J , 5

##### Diagnosis.

**Figure 5.**
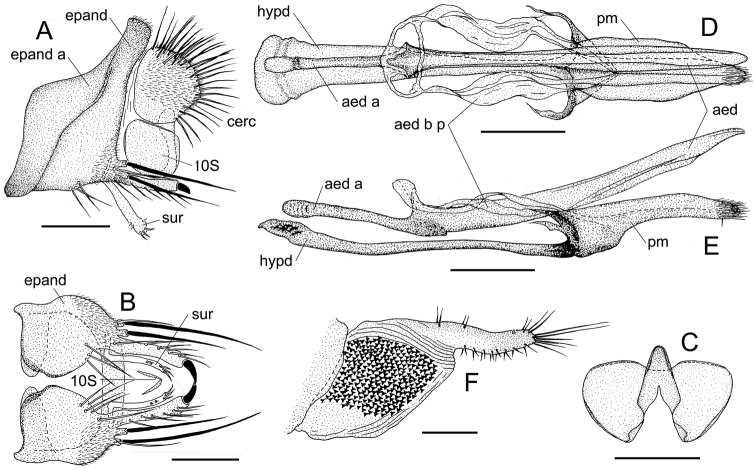
*Colocasiomyia liae* Jiao & Gao, sp. nov. Adult male (holotype #10485) and female (paratype, #10486) from Qimaba, Lüchun, Yunnan, China. **A** periphallic organs (lateral view) **B** periphallic organs except cerci (ventral view) **C** tenth sternite (posteroventral view) **D** phallic organs (dorsal view) **E** phallic organs (lateral view) **F** oviscapt (lateral view). Abbreviations: aed = aedeagus, aed a = aedeagal apodeme, aed b p = aedeagal basal process, cerc = cercus, epand = epandrium, epand a = epandrial apodeme, hypd = hypandrium, pm = paramere, 10S = tenth sternite. Scale lines: 0.1 mm.

This species closely resembles *C. rhaphidophorae* in external morphology and structure of male and female genitalia, but can be distinguished from the latter by epandrial ventral lobe rod-like, distally slightly broadened in lateral view, apically inlaid with a relatively long, claw-like peg (Fig. 5A, B); aedeagus broader in distal half in ventral view (Fig. 5D); surstylus apically expanded, with 1 dorsosubapical, 2 ventrosubapical, minute sensilla in addition to three small setulae at apex (Fig. 5A); distal, narrow elongation of oviscapt somewhat sinuate in lateral view (Fig. 5F).

##### Description.

(♂, ♀). ***Head***: Supracervical setae about 9−10 per side. Dorsomedial arm of tentorial apodeme about 1/3 as long as dorsolateral arm. Eye red, somewhat roundish, with very sparse interfacetal setulae. Frontal vitta mat, black. First flagellomere not concave on inner margin. Facial carina broad trapeziform, medially twice as wide as first flagellomere, as long as pedicel and first flagellomere combined. Palpus convex on ventrodistal portion. Cibarium posterior sensilla minute, 2 or 3 per side. Labellum with 34 pseudotracheae per side.

***Thorax*** (Fig. 3F, H): Scutum, scutellum and thoracic pleura glossy, black. Acrostichal setulae in 6 rows.

***Wing*** (Fig. 3I) hyaline, veins yellow. Halter grayish brown except for grayish yellow stalk.

***Legs*** (Fig. 3F, J) blackish brow to black except for grayish yellow knee joints and tarsi: Foreleg second tarsomere with 10 pegs (Fig. 3J). Foreleg coxa large, with 1−2 long setae on underside near attachment to trochanter. Small preapical dorsal seta present only on hindleg tibiae.

***Abdomen*** (Fig. 3F): Tergites glossy, entirely black except for anterior, narrow, grayish margins on III−VI; II to VI+VII each bearing setulae and setae in approximately 3−4 transverse rows; setae of posteriomost row largest. Sternites yellowish brown to blackish brown; VI posteriorly bilobed.

***Male terminalia*** (Fig. 5A−E): Epandrium dorsally narrow, with prominent apodeme on anteromedial to ventral margin, unpubescent on medial and anteroventral portions; ventral portion curved inward, apically articulated to lateral arm of hypandrium; ventral lobe well developed, with two long and one medium-length, thick setae on its insertion, and 6−7 setae along ventral margin (Fig. 5A, B). Cercus somewhat trapeziform, pubescent on dorsal 2/3, with ca 29 setae on dorsal 1/3 and posterior margin (Fig. 5A). Surstylus entirely narrow sclerite, grayish yellow, elongated downward, basally articulated with epandrial ventral lobe (Fig. 5A, B). Tenth sternite medially forms vertical ridge, ventrally folded upwardly, forming a large, peripheral lobe (Fig. 5C). Hypandrium long, thin plate, distal 1/2 constricted, posteriorly T-shaped, with lateral arms fused to aedeagal basal processes (Fig. 5D, E). Paramere broad, double-layered, coalescent to hypandrium, gently curved ventrad at distal 1/3, ventrosubapically with a minute sensillum, distally hirsute (Fig. 5D, E). Aedeagus entirely unpubescent, bent dorsad gently (Fig. 5D, E); aedeagal basal processes somewhat membranous, connecting dorsobasal corners of aedeagus and lateral arms of inner and outer layers of hypandrium (Fig. 5D, E).

***Female terminalia*** (Fig. 5F): Tergite VII mid-dorsally not constricted; VIII pubescent nearly entirely, with 3 setae in a vertical row on discolored, posteroventral portion. Oviscapt with distal, narrow elongation in addition to proximal, broad portion; proximal portion with large patch of dense, distinct warts; distal, narrow portion, with ca 2, 8 and 5 trichoid ovisensilla per side on basal 1/3 of dorsal margin, entire ventral margin, and at apex, respectively, and a tiny, peg-like ovisensillum near subapical, dorsal margin.

***Measurements***: BL = 2.45 (1♀ paratype: 2.60) mm, ThL = 1.10 (0.93) mm, WL = 2.08 (1.80) mm, WW = 0.91 (0.75) mm.

***Indices***: arb = 0/0 (1♀ paratype: 0/0), FW/HW = 0.57 (0.58), ch/o = 0.47 (0.51), prorb =1.14 (1.05), rcorb = 0.28 (0.45), orbito = 0.67 (0.86), vb = 0.34 (0.35), dcl = 0.53 (0.53), dcp = 0.97 (0.96), sterno = 0.75 (0.68), sctl = 0.56 (0.59), sctlp = 1.20 (1.03), C = 1.75 (1.64), 4c = 1.12 (1.18), 4v = 1.58 (1.70), 5x = 0.88 (0.92), ac = 3.94 (3.68), M = 0.36 (0.34), C3F = 0.82 (0.74).

##### Material examined.

***Holotype*** ♂ (#10485): China: *ex* inflorescence of *Rhaphidophora crassicaulis* Engl. & Krause, Qimaba, Lüchun, Yunnan, China, 22°48.0'N, 102°15.0'E, ca 750 m, 6.vii.2020, Jian-Jun Gao and Run-Jie Jiao (KIZ). ***Paratype***: same data as holotype (1♀: #10486) (KIZ).

##### Distribution.

China (Yunnan).

##### Host plants.

*Rhaphidophora crassicaulis* Engl. & Krause (Fig. 1E–H).

##### Etymology.

Patronym, in honor of Professor Heng Li (Kunming Institute of Botany, Chinese Academy of Sciences), who helped us with the identifications of various aroid host plants of *Colocasiomyia* flies.

##### Remarks.

Numbers of adults of this species were obtained by rearing infructescences of *R. crassicaulis* collected on November 1, 2018 from the type locality, indicating that this species breeds on inflorescences/infructescences of this host plant. These adults were not defined as type specimens due to obviously insufficient body pigmentation and sclerotization, though some of them were used for DNA barcoding.

## Supplementary Material

XML Treatment for Colocasiomyia gigantea

XML Treatment for Colocasiomyia todai

XML Treatment for Colocasiomyia liae
